# Development and proof-of-concept of a multicenter, patient-centered cancer registry for breast cancer patients with metastatic disease—the “Breast cancer care for patients with metastatic disease” (BRE-4-MED) registry

**DOI:** 10.1186/s40814-019-0541-3

**Published:** 2020-02-04

**Authors:** Stephanie Stangl, Kirsten Haas, Felizitas A. Eichner, Anna Grau, Udo Selig, Timo Ludwig, Tanja Fehm, Tanja Stüber, Asarnusch Rashid, Alexander Kerscher, Ralf Bargou, Silke Hermann, Volker Arndt, Martin Meyer, Manfred Wildner, Hermann Faller, Michael G. Schrauder, Michael Weigel, Ulrich Schlembach, Peter U. Heuschmann, Achim Wöckel

**Affiliations:** 1 Institute of Clinical Epidemiology and Biometry (ICE-B), University of Würzburg, Würzburg, Germany; 2 Department of Gynecology and Obstetrics, University Hospital Würzburg, Würzburg, Germany; 3 Department of Obstetrics and Gynecology, Heinrich-Heine-University Düsseldorf, Düsseldorf, Germany; 4 Zentrum für Telemedizin Bad Kissingen (ZTM), Bad Kissingen, Germany; 5 Comprehensive Cancer Center Mainfranken, University Hospital Würzburg, Würzburg, Germany; 6 Clinical Cancer Registry Lower Franconia, Würzburg, Germany; 7 Epidemiological Cancer Registry Baden-Württemberg, German Cancer Research Center, Heidelberg, Germany; 8 Centre of Early Cancer Detection and Cancer Registration, Bavarian Health and Food Safety Authority, Nürnberg, Germany; 9 Bavarian Health and Food Safety Authority, Munich, Germany; 10 Department of Medical Psychology and Psychotherapy, Medical Sociology and Rehabilitation Sciences, University of Würzburg, Würzburg, Germany; 11 Clinic of Gynecology and Obstetrics, Hospital Aschaffenburg-Alzenau, Aschaffenburg, Germany; 12 Clinic for Gynecology and Obstetrics, Hospital Leopoldina Schweinfurt, Schweinfurt, Germany; 13 Clinic of Gynecology, Caritas Hospital Bad Mergentheim, Bad Mergentheim, Germany; 14 Center for Clinical Studies, University Hospital Würzburg, Würzburg, Germany; 15 Comprehensive Heart Failure Centre, Würzburg, Germany

**Keywords:** Metastatic breast cancer, Patient-centered registry, Patient’s needs, m-Health, Health care service research

## Abstract

**Background:**

Patients with metastatic breast cancer (MBC) are treated with a palliative approach with focus on controlling for disease symptoms and maintaining high quality of life. Information on individual needs of patients and their relatives as well as on treatment patterns in clinical routine care for this specific patient group are lacking or are not routinely documented in established Cancer Registries. Thus, we developed a registry concept specifically adapted for these incurable patients comprising primary and secondary data as well as mobile-health (m-health) data.

**Methods:**

The concept for patient-centered “Breast cancer care for patients with metastatic disease” (BRE-4-MED) registry was developed and piloted exemplarily in the region of Main-Franconia, a mainly rural region in Germany comprising about 1.3 M inhabitants. The registry concept includes data on diagnosis, therapy, progression, patient-reported outcome measures (PROMs), and needs of family members from several sources of information including routine data from established Cancer Registries in different federal states, treating physicians in hospital as well as in outpatient settings, patients with metastatic breast cancer and their family members. Linkage with routine cancer registry data was performed to collect secondary data on diagnosis, therapy, and progression. Paper and online-based questionnaires were used to assess PROMs. A dedicated mobile application software (APP) was developed to monitor needs, progression, and therapy change of individual patients. Patient’s acceptance and feasibility of data collection in clinical routine was assessed within a proof-of-concept study.

**Results:**

The concept for the BRE-4-MED registry was developed and piloted between September 2017 and May 2018. In total *n* = 31 patients were included in the pilot study, *n* = 22 patients were followed up after 1 month. Record linkage with the Cancer Registries of Bavaria and Baden-Württemberg demonstrated to be feasible. The voluntary APP/online questionnaire was used by *n* = 7 participants. The feasibility of the registry concept in clinical routine was positively evaluated by the participating hospitals.

**Conclusion:**

The concept of the BRE-4-MED registry provides evidence that combinatorial evaluation of PROMs, needs of family members, and raising clinical parameters from primary and secondary data sources as well as m-health applications are feasible and accepted in an incurable cancer collective.

## Background

Breast cancer (BC) is the most common cancer in women in Germany and worldwide [1, 2]. Metastatic breast cancer (MBC) is incurable with a median survival time for patients between 2 and 4 years [3]. Treatment of BC patients is a multidisciplinary approach since multiple health care professionals (e.g., psychologist, physician) as well as several disciplines of physicians (e.g., oncologist, gynecologist) are involved. There are national and international clinical guidelines with evidence- and consensus-based recommendations for guiding treating physicians on effective therapies [4–6]. However, international studies report frequent deviations from recommendations of existing guidelines [7, 8]. Identification of reasons and obstacles of treatment heterogeneity can provide insights on individual-patient and on structural level. The Cancer Registries in Germany provide individual information on diagnosis, therapy, and outcome, but information on patient-reported outcomes (PROs) for MBC patients are lacking [9]. Potential obstacles, which might lead to deviations from guideline recommendations, are only described for patients with early BC but not for patients with MBC in Germany, yet [10, 11]. In Germany, health care is divided into the distinct sectors acute care, rehabilitation care, and outpatient care, with different facilities being responsible for covering the costs. Thus, providing appropriate BC care throughout the sectors for MBC patients might be specifically challenging in this setting.

Therefore, the concept of a multicenter, patient-centered registry specifically adapted to the needs of patients with metastatic BC was developed. The BRE-4-MED registry aims to combine data on clinical parameters from treating physicians and established regional Cancer Registries with information on met and unmet needs reported by the patients themselves and their family members during the course of disease. Data collection and record linkage were piloted within a proof-of-concept study. Furthermore, patients’ acceptance of m-health applications, including APP-based or web-based questionnaires, was also tested to provide reliable information in patients with MBC. This paper describes the concept of the BRE-4-MED registry and gives results of the proof-of-concept study.

## Methods

In 2016, the German Federal Ministry of Education and Research (BMBF) invited proposals for developing concepts on “Aufbau modellhafter Register für die Versorgungsforschung” (i.e., development of exemplary registries for health care service research). The proposed *Bre*ast cancer care *for* patients with *me*tastatic *d*isease (BRE-4-MED) registry was one of 16 registries funded within the conception phase from September 2017 to May 2018. During these 9 months, the planned BRE-4-MED concept was developed. In addition, a proof-of-concept study was carried out to assess feasibility of the concept.

### Objectives of the BRE-4-MED registry

The BRE-4-MED registry aims (1) to evaluate currently administered therapies in clinical routine after first diagnosis of MBC considering existing guidelines; (2) to identify barriers hampering guideline implementation in clinical routine care on organizational (e.g., rural versus urban region) or individual (e.g., patient’s age) level in different health care settings (e.g., hospital care, outpatient care); (3) to document met and unmet needs of patients and their family members over the entire course of the disease.

The primary aim is to assess prevalence of guideline-adherent therapies operationalizing quality indicators based on therapy recommendations of evidence- and consensus-based National Breast Cancer Guideline [12, 13]. Secondary aims are the association of guideline-adherent therapy of MBC patients with overall and progression-free survival as well as PROs like the following: quality of life, physical function, depression, and anxiety.

### Development of quality indicators

Possible quality indicators were identified through the national evidence- and consensus-based guideline on breast cancer [4]. The standardized process followed criteria of the First Scientific Forum on Assessment of Quality of Care and Outcome Research in Cardiovascular Disease and Stroke of the American Heart Association as well as the requirements for clinical performance measures according to the German healthcare system, which were also used for previous development of performance measures in Deep Brain Stimulation in patients with Parkinson’s Disease [14–16]. The assessment of practicability and relevance of the proposed quality indicators was carried out in a moderated meeting of the Scientific Advisory Board.

### Source population and setting

**Fig. 1:**
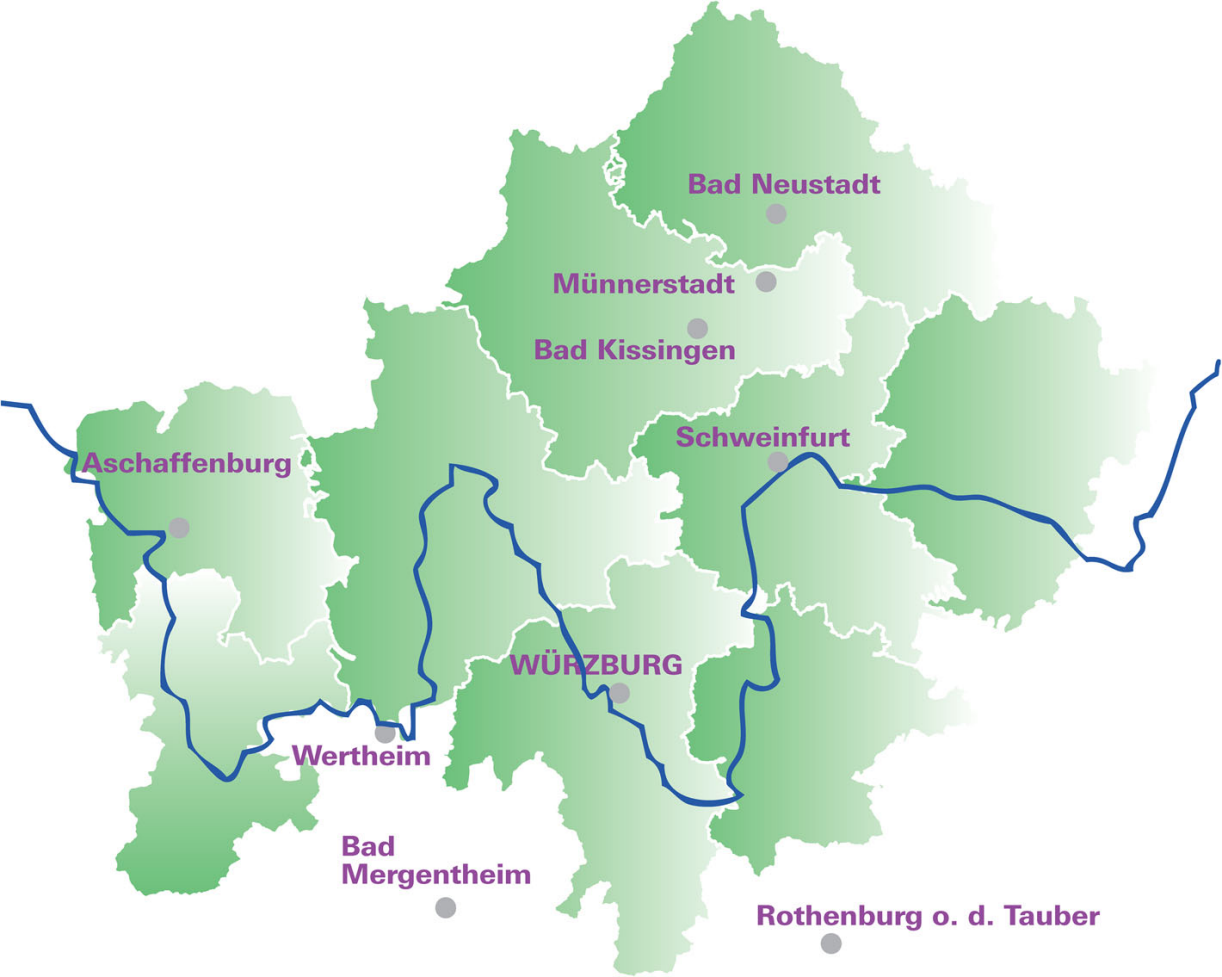
Map of Lower Franconia and the Main-Tauber-Kreis (Baden-Württemberg) lying in the south west of Würzburg

The source population of the BRE-4-MED registry is the region of Main-Franconia, Germany, which comprises the northwest of Bavaria (Lower Franconia) and the northeast of Baden-Württemberg (Main-Tauber-Kreis) with about 1.3 M inhabitants (Fig. 1). Main-Franconia represents urbanized counties (e.g., Würzburg, Aschaffenburg), and rural parts (e.g., Schweinfurt, Kreis Main-Tauber). In the region, approximately 1160 patients are diagnosed with BC per year based on data from the Clinical Cancer Registry Lower Franconia; it is estimated that of those, about 205 patients (male and female) suffer from metastatic disease.

### Main-Franconia as model region

The study region, Main-Franconia, was chosen as particularly suitable for addressing the aims of BRE-4-MED since it allows to study the influence of urban and rural areas in health care provision as well as challenges of border-crossing care between different federal states (Bavaria and Baden-Württemberg). Main-Franconia also provides an excellent structure of existing and successfully cooperating networks and partners like the Comprehensive Cancer Centre Main-Franconia (CCC MF). The BRE-4-MED registry comprises the partners being involved in providing or evaluating health care such as (1) CCC MF; (2) Department of Gynecology at the University Hospital of Würzburg as coordinating center; (3) Institute of Clinical Epidemiology and Biometry (ICE-B) of the University Würzburg as method center; (4) Cancer Registries of Bavaria and Baden-Württemberg for provision of secondary data on diagnosis, therapy, and progression of MBC care; (5) Centre for Telemedicine for developing and hosting web-based application; (6) specialized acute care hospitals for patient recruitment; (7) ambulatory health care centers and outpatient physicians for reporting on follow-up care of recruited patient. These comprehensive partners enable to connect patient-reported information (primary data) and clinical information (secondary data) from multiple overlapping sources at different time points within one registry. Structures of BRE-4-MED, successfully established within the model region of Main-Franconia, can be transferred on national or international level.

### Participating hospitals

Four hospitals providing breast cancer care for the region of Main-Franconia participate in the BRE-4-MED registry. Three of them are located in Bavaria (University Hospital Würzburg, Leopoldina-Hospital Schweinfurt, and Hospital Aschaffenburg-Alzenau) and one (Caritas-Hospital Bad Mergentheim) is located in Baden-Württemberg. All participating hospitals are BC centers certified by the German Cancer Society (DKG). About 80% of the MBC patients of the region are diagnosed in these hospitals (unpublished data of the Cancer Registry of Lower Franconia).

### Inclusion and exclusion criteria

BRE-4-MED enrolls patients, both sexes, consecutively diagnosed with MBC, 18 years or older, and who gave written informed consent to participate. Exclusion criteria are minimized to age (< 18 years) and disease (non-MBC) to guarantee a most representative study population of clinical routine care.

### Data and survey procedures

**Fig. 2:**
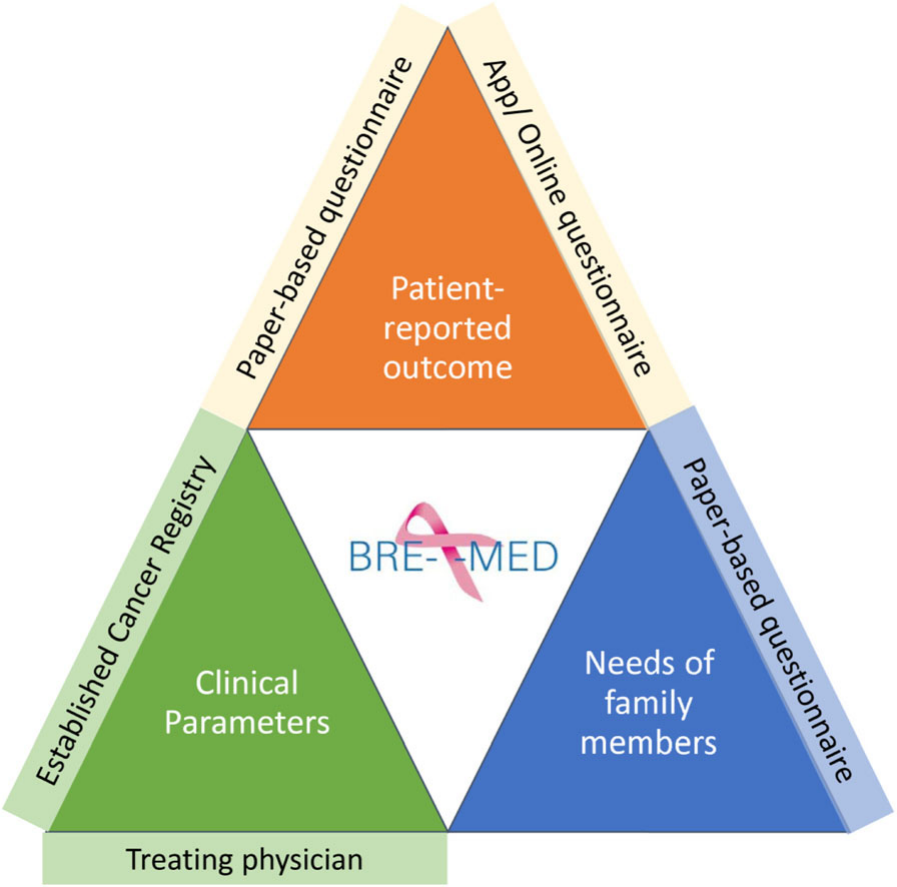
Overview of sources of primary and secondary data of the BRE-4-MED registry

The BRE-4-MED registry combines data from first diagnosis of MBC up to 18 months from multiple overlapping sources such as patients and their family members (primary data), treating physician, and Cancer Registries (secondary data). National and international accepted classification systems and instrumental scales validated for use in Germany are selected [17, 18]. Table 1 provides an overview of items assessed at baseline and follow-up. Figure 2 provides an overview on data sources utilized for the BRE-4-MED registry.

**Table 1:** Overview of endpoints, time of data collection for the BRE-4-MED registry concept

Observation	Screening	enrolment/baseline	3 months	6 months	12 months	18 months (study termination)	APP (Fort-nightly)	Cancer registry data	Occurrence of event
Timeframe		+ 1 day	+/− 2 weeks	+/− 2 weeks	+/− 2 weeks	+/− 2 weeks	+/− 3 days	+/− 1 month	
Eligibility check	✓	✓							
Patient information	✓								
Informed consent		✓							
Endpoints:									
(a) Treatment:									
Physicians questionnaire		✓						✓	✓
(b) Patient and caregiver:									
Quality of life/health status^1^		✓	✓	✓	✓	✓	✓		
Physical functioning^2^		✓	✓	✓	✓	✓	✓		
Depression/anxiety^3^		✓	✓	✓	✓	✓			
Caregiver burden^4^			✓	✓	✓	✓			
Individual patient’s needs^5^		✓	✓	✓	✓	✓	✓		
Access to health care services^6^		✓	✓	✓	✓	✓	✓		
Physician’s empathy^7^		✓	✓	✓	✓	✓			
Vital status, therapy change, progression		✓	✓	✓	✓	✓	✓		
Socio-demographics		✓	✓	✓	✓	✓			
(3) Treatment framework:									
Living area		✓							
Health care settings		✓							✓
Core Data	✓	✓						✓	
Confounders		✓						✓	✓
Endpoints		✓	✓	✓	✓	✓			

^1^EORTC-QLQ-2 (Version 3); ^2^PROMs (PROMIS, physical function) 4-item scale; ^3^depression and anxiety (Patient Health Questionnaire-4 (PHQ-4)); ^4^caregiver burden (Caregiver Reaction Assessment (CRA)); ^5^patient’s health care needs (i.e., social, informational, psycho-oncological, administrative support); ^6^access to health care services (i.e., psycho-oncological therapy, self-aid group, medical rehabilitation, palliative care, sports); ^7^consultation and relational empathy (CARE)

### Patients and family members

Patients diagnosed with MBC are informed by the project staff (i.e., treating physician, study nurse) about the BRE-4-MED registry and asked to participate. The participants fill out a comprehensive questionnaire at baseline (i.e., diagnosis of metastatic disease) and at 3, 6, 12, and 18 months after inclusion into the registry. To obtain high response rates, a predefined algorithm is used including regular postal and phone contact. The paper-based follow-up questionnaire is sent at follow-up time points by mail to the study participants with a cover letter and a postpaid return envelope. The letter also includes an anonymous paper-based questionnaire for the caregiver that the patient hands over to a family member in case she/he is caring for the patient. If the follow-up questionnaire is not returned to the ICE-B within 14 days, the participant is contacted by phone up to three times and a telephone interview by trained study personnel is offered to the patient. Finally, in case of lacking contact, the local registration office is contacted to validate vital status or change of address.

### PROs documentation by m-health solutions

**Fig. 3:**
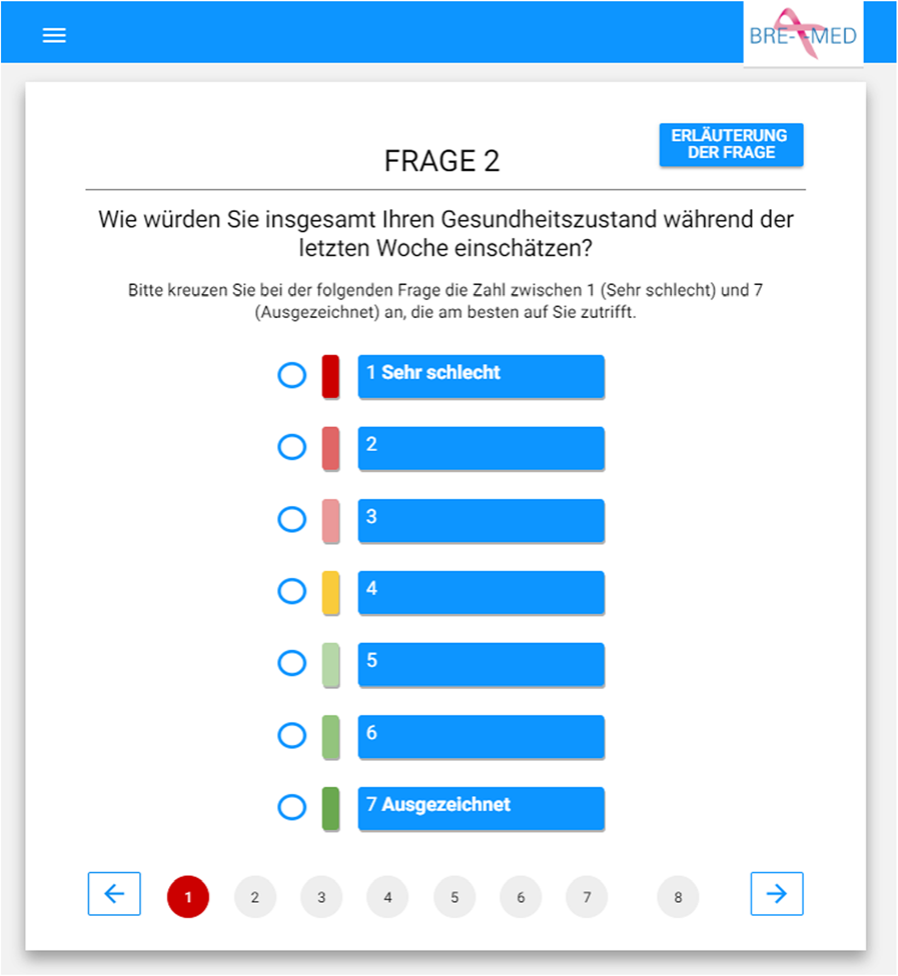
Screenshots of the BRE-4-MED patient App: Question 2 on health status (European Organisation for Research and Treatment of Cancer–Quality of Life-2 (EORTC-QL-2 (Version 3)). The scale comprises answer options from “very bad” (1) to “excellent” (7). The red circle at the bottom gives an overview of which questions have not been answered yet (red circle) and which questions have already been answered (green circle: not shown in this screen shot). In the right upper part of the screenshot is a button “Erläuterungen der Frage”: the patient can click on it to see more information on how the question is meant

For the BRE-4-MED registry an APP and Online Questionnaire was developed by the Centre for Telemedicine (ZTM, Bad Kissingen, Germany) to test the acceptance of m-health solutions for outcome documentation in incurable patients [19]. After finalizing the APP, patients were offered to voluntarily use the APP or a web-based version of the questionnaire and were guided in installing on his/her private mobile device at baseline. The APP or web-based questionnaire is filled out on a regular basis (e.g., every 2 weeks). The password-secured APP sends a regular alert function to remind the patients of filling in the short questionnaire. As an alternative, patients can choose to use a web-based online-questionnaire instead. With the APP or online questionnaire, the patient can report information on progression of therapy chance as well as quality of life, physical function, and met/unmet needs within shorter intervals (e.g., fortnightly) than the paper-based follow-up (i.e., baseline and 3, 6, 12, and 18 month later). Figure 3 provides a screenshot of the APP. Information of burden of caregiving is obtained from the carer, which is contacted via the follow-up mail to the patient. The BRE-4-MED participant is asked to forward the questionnaire to a relative of her/his choice. No person-identifiable data are assessed from the relative.

### Physician

The treating physician enters information on diagnosis, guideline conformity, and comorbidities of the patient into an electronic Case Record Form (eCRF). At occurrence of a progression or therapy change during the course of disease, the physician is asked to give information on therapy, diagnosis, and guideline conformity again. By gaining information on progression and therapy change from several overlapping sources of information, e.g., the patient (paper-based Case Record Form (pCRF) and APP) and Cancer Registries, it is possible to contact the respective physician to fill out another questionnaire.

### Testing ways of record linkage of m-health reported data and data from Cancer Registries with paper-based reported PROs

**Fig. 4:**
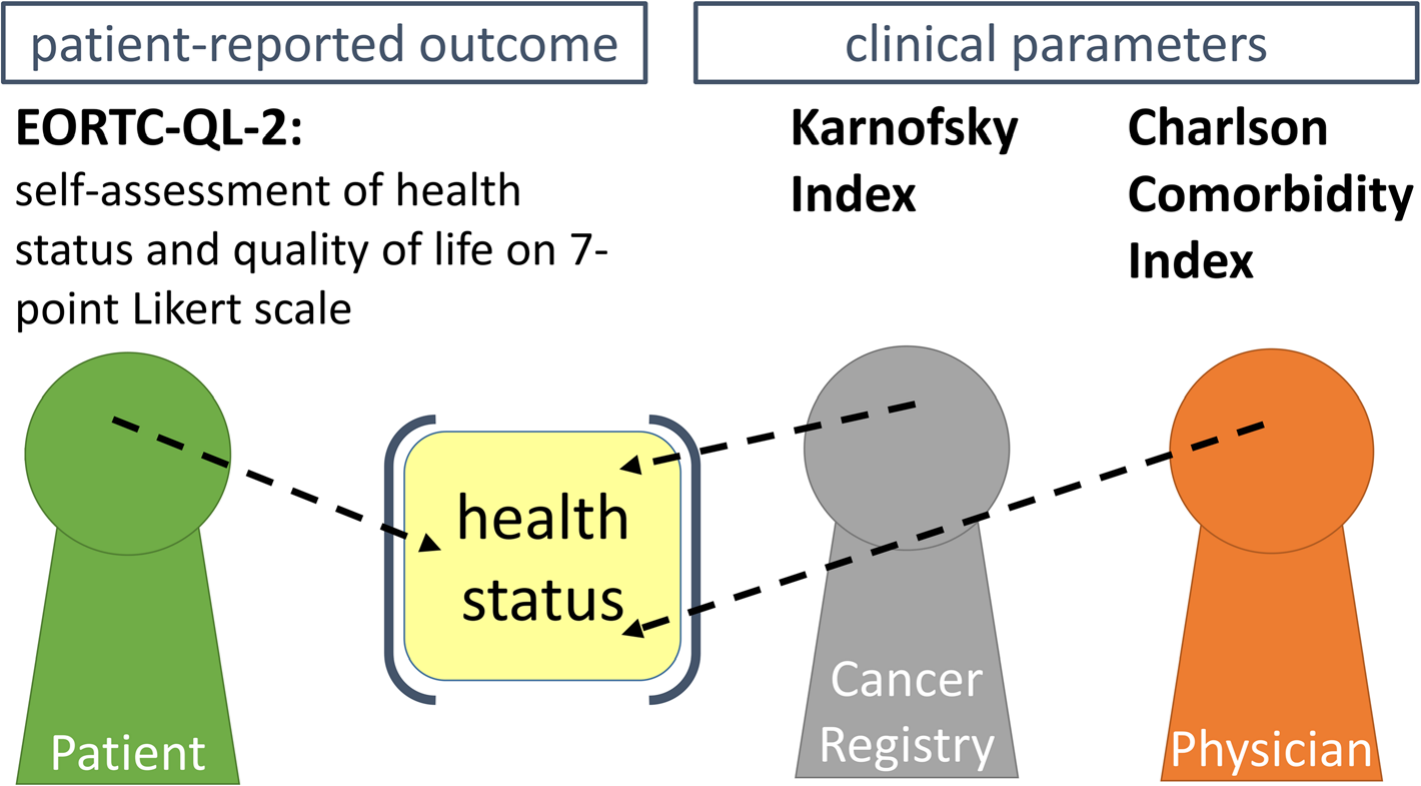
Concept of utilizing different sources on outcome evaluation (as example serves health status)

Cooperation was established with the responsible cancer registries for Main-Franconia (Clinical Cancer Registry of Lower Franconia and Cancer Registry Baden-Württemberg) to match information from the BRE-4-MED participants with information reported to the Cancer Registries. For retrospective crosschecks of quality and completeness of data regarding diagnosis, therapies, and outcome of patients recruited in the BRE-4-MED registry, a regular record linkage with data from the Cancer Registries in Bavaria and Baden-Württemberg is planned. The feasibility of the record linkage was tested in the pilot phase. Figure 4 gives an overview how different perspectives are utilized for BRE-4-MED.

### Governance and patient involvement

A steering committee and a scientific advisory board (SAB) were created to provide project and research guidance and support on all aspects of the development, implementation, and management of the registry. The BRE-4-MED registry closely collaborates with the regional patient advocacy group “Womens’ self-aid after cancer” (“Frauenselbsthilfe nach Krebs”) for establishing and running the registry, developing patient information material.

### Rationale of the proof-of-concept study (March to May 2018)

To assess the feasibility and acceptance of the standardized data collection, data management, and questionnaires (paper- and web-based) of patients and physicians as well as record linkage with Cancer Registries, a proof-of-concept study was conducted. Table 2 provides an overview of feasibility criteria assessed in the proof-of-concept study. All four participating hospitals were asked to enroll at least one patient with MBC. To ensure that data of the patient were already available in the Cancer Registries also patients with prevalent MBC were eligible for the pilot study. Patients were offered to test the voluntary BRE-4-MED APP or web-based questionnaire. The included patients were followed up after 1 month by paper-based questionnaires. The proof-of-concept study revealed first preliminary data on met and unmet needs of patients with MBC in Main-Franconia.

**Table 2:** Overview of a priori–defined criteria regarding feasibility of the developed BRE-4-MED concept

Defined feasibility criteria for proof-of-concept study • Linkage of routine data from established Cancer Registry with patient-reported outcomes: combining secondary and primary data ○ Can patients, who gave their consent to data linkage, be identified in the established Registries? ○ Is data exchange between method center (ICE-B), trusted third parties and Cancer Registries practicable? • Usage of m-health devices by patients and treating physicians ○ Does the concept (log in, username, completion of questionnaire, and transfer to server/central database) work out? • Proof of organizational requirements regarding: ○ Transfer of written informed consent to method center (ICE-B) for follow-up procedures ○ Procedures of central follow-up by method center feasible (ICE-B) (postal and phone) • Acceptance of questionnaires/m-health devices in an incurable cancer collective as well as by clinical staff ○ Usage of m-health device ○ Fill out/return of questionnaire/completeness of documentation (appropriateness and comprehensibility of questions in an incurable cohort)	

### Ethics committee and data protection approval

The registry and the proof of concept study were approved by the ethics committee of the Medical Faculty University Hospital Würzburg (no. 245/17), the Medical Faculty of the University of Heidelberg (S-223/2018), and the Physicians’ Chamber of Baden-Württemberg (B-F-2018-034). The data management concept of the registry was approved by the corresponding data protection officer (DS-117.605-17/17). BRE-4-MED is registered within the German Clinical Trials Register (DRKS): DRKS-ID DRKS00013726 (registered: 6 February 2018).

## Results

The whole process of developing and piloting the registry concept took place from September 2017 to May 2018. The concept was presented and discussed during the steering committee meetings. In total, two steering committee and two Scientific Advisory Board meetings took place during the whole process for informing the development of the registry and the pilot phase.

### Piloting the methodology of the BRE-4-MED registry within a proof-of-concept study

A proof-of-concept study was performed in all four participating hospitals to assess the feasibility of the developed concept with all questionnaires, way of data exchange, and record linkage with the Cancer registries. In addition, patient’s acceptance to use the BRE-4-MED APP or online questionnaire was investigated.

Patients with prevalent or newly diagnosed MBC were eligible for the pilot study. Overall, *n* = 31 patients gave written informed consent to be included in the BRE-4-MED pilot study testing baseline assessment and 1-month follow-up. The University Hospital Würzburg, as breast center in Lower Franconia with the highest volume of treating MBC patients, served as model to assess participation rate: *N*_Würzburg_ = 22 (36%) patients were included of *n*_Würzburg_ = 61 eligible patients with prevalent and newly diagnosed MBC asked. Documented reasons for non-participation were “others,” “does not want to,” or “is not able to.”

### Baseline assessment

A total of *n* = 31 patients from all four participating hospitals provided informed consent. The mean age of the participants was 57.1 (± 13.5) years and *n* = 30 (97%) were female. *N* = 30 (97%) patients filled out a baseline questionnaire. The BRE-4-MED participant with the longest metastatic course was diagnosed in January 2004, and the patient with the shortest received the MBC diagnosis in March 2018. Table 3 shows baseline characteristics of participants.

**Table 3:** Patient characteristics at baseline

BRE-4-MED participants with baseline information	
Female sex, *n* (%)	30 (96.8)
Age, mean (std), years	57.1 (± 13.5)
Time since diagnosis of MBC, median (IQR), months	21.0 (7–45)
Care given by family member*, *n* (%)	6 (21.4)
Returned questionnaires of family members*, *n* (%)	15 (50.0)
Number of metastasis location, *n* (%)	
1	9 (29.0)
2	10 (32.3)
3	5 (16.1)
4	5 (16.1)
5	1 (3.2)
6	1 (3.2)
Kind of administered medication, *n* (%)	
No information on medication specified	1 (3.2)
Oral only	2 (6.5)
Oral + subcutaneous	1 (3.2)
IV only	14 (45.2)
IV + oral	11 (35.5)
IV + oral and subcutaneous	2 (6.5)

*Analyses were restricted to patients without missing values

### Follow-up assessment

*N* = 23 (77%) participants provided information at 1-month follow-up (*n* = 1 death, *n* = 22 alive). Reasons for non-replying were declining health condition (*n* = 1) and refusal to further participate (*n* = 6). *N* = 15 relatives’ questionnaires on caregiver burden were filled out and sent back but only *n* = 6 (21%) patients stated explicitly that they were cared by a family member.

### Acceptance of questionnaires

The eCRFs were evaluated positively by the treating physicians due to its intuitive design and uncomplicated and secured online transfer to the method center. The patient’s questionnaire was pre-tested by a patient representative and also evaluated positively and relevant to patients themselves.

### Acceptance to m-health solutions

Furthermore, *n* = 23 (missing *n* = 8) participants indicated to own a smartphone or tablet and *n* = 21 (missing *n* = 10) own a computer with internet access. The APP or online questionnaire was filled out by *n* = 7 patients at baseline and *n* = 2 at follow-up after 2 weeks. Feedback of the recruiting sites’ regarding the usage of the m-health tools referred mainly to initial technical problems with the APP or online-questionnaire due to highly secured firewalls of the hospitals, which could be solved by in assistance with the ZTM. The mean age of patients utilizing m-health applications was 49.2 (± 10.2) years.

### Record linkage with Cancer Registry data

**Fig. 5:**
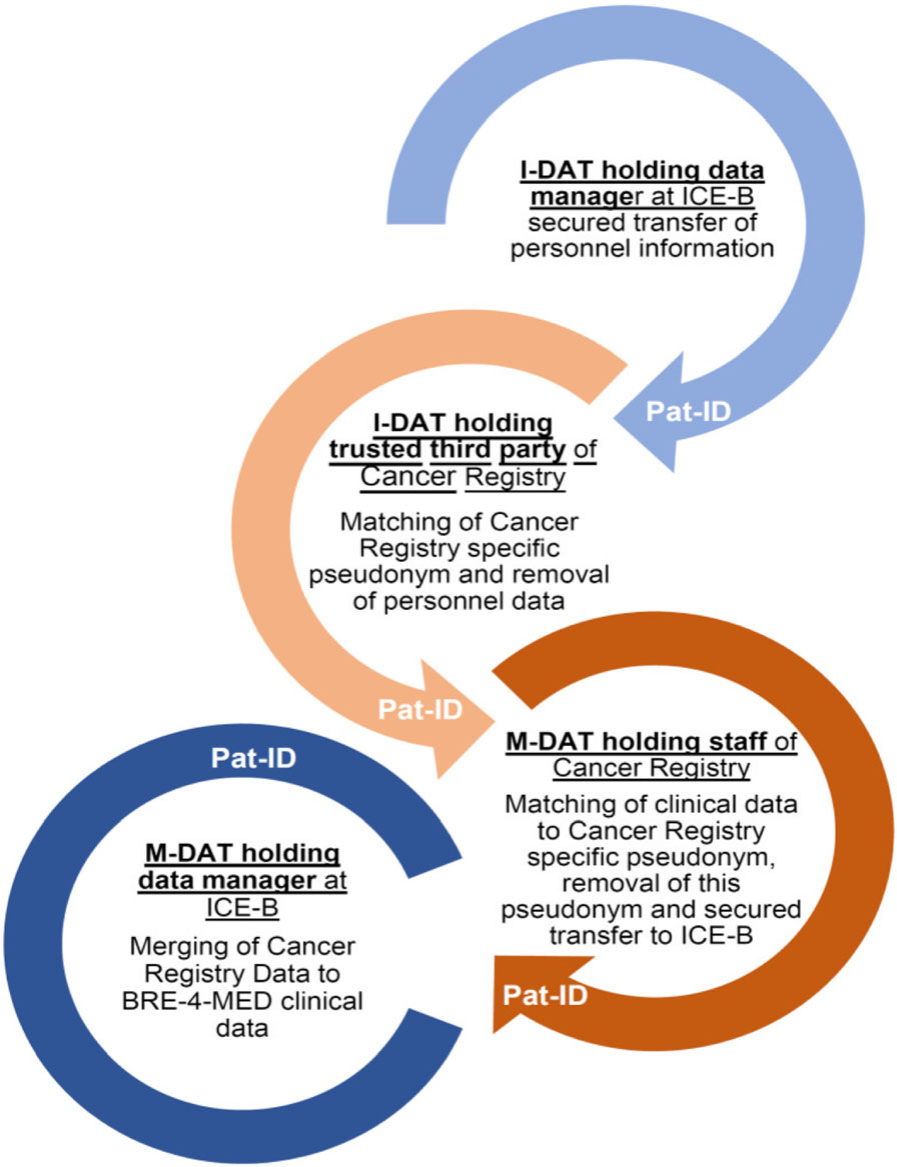
Record linkage process with separation between identifiable (I-DAT) and medical data (M-DAT) at the processing sites (ICE-B, Cancer Registries)

The record linkage concept with the established Cancer Registries of Bavaria and Baden-Württemberg was approved by the competent data protection officers of the registries. For record linkage the Cancer Registries and their trusted third parties, as holder of patient-identifiable data, are involved in the process. Figure 5 gives an overview of the described process. For *n* = 17 BRE-4-MED participants information was available in the Cancer Registries and record linkage was carried out successfully. Information on Karnofsky Index was available in *n* = 10 (59%) patients (Table 4).

**Table 4:** Information on health status at baseline from different sources

Health status	
Reported by physician: Charlson Comorbidity Index, *n* (%)	
6	28 (87.5)
7	2 (6.3)
13	1 (3.1)
Reported by Cancer Registry: Karnofsky Index, *n* (%)	
90–100%	7 (22.6)
70–80%	3 (9.7)
No information on Karnofsky Index in Cancer Registries available	7 (22.6)
missing	14 (45.2)
45.2)Self-rated quality of life (EORTC-QL-2 (Version 3)) by *n* = 30 patients (pCRF): 7-point Likert Scale, mean (std)	4.9 (± 1.2)
Self-rated quality of life (EORTC-QL-2 (Version 3)) by *n* = 7 patients (APP, eCRF): 7-point Likert Scale, mean (std)	5.9 (± 0.9)
Consistency of self-reported quality of life between pCRF and APP at baseline (*n* = 7), Pearson correlation coefficient	0.90
Self-rated health status (EORTC-QL-2 (Version 3)) by *n* = 30 patients (pCRF): 7-point Likert Scale, mean (std)	4.8 (± 1.2)
Self-rated health status (EORTC-QL-2 (Version 3)) by *n* = 7 patients (APP, eCRF): 7-point Likert Scale, mean (std)	5.6 (± 1.3)
Consistency of self-reported health status between pCRF and APP at baseline, Pearson correlation coefficient	0.96

### Access to appropriate health services structures

The BRE-4-MED registry comprises rural and urban areas. Participants were asked about access to pre-defined appropriate health care providers/institutions or to specify as free text, if any unmet need was identified. Overall, the majority of pilot study participants stated that they had either no problem to access health care services such as “physiotherapy,” “general practitioner,” “medical specialist,” and “rehabilitation centers” or that the access to structures as “palliative care” and “nutritional counseling” was not necessary for them. Still a minority (*n* ≤ 5) of MBC patients in the pilot study had no access to “palliative care” or “psychotherapy” (see Table 5).

**Table 5:** Patient-reported access to health care service providers regarding breast cancer

*N* = 30 CRFs at baseline			
Access to:	No problem	No access possible	Not necessary
	*n* (%)		
General practitioner	27 (93.1)	0 (0)	2 (6.9)
Medical specialist	30 (100)	0 (0)	0 (0)
Follow-up care	16 (69.6)	2 (8.7)	5 (21.7)
Psychotherapy	8 (27.6)	3 (10.3)	18 (62.1)
Psycho-oncologist	7 (25.9)	2 (7.4)	18 (66.7)
Cancer counseling centers	7 (24.1)	4 (19.8)	18 (62.1)
Self-aid groups	7 (25.9.)	2 (7.4)	18 (66.7)
Physiotherapy	24 (85.7)	1 (3.6)	3 (10.7)
Nutritional counseling	11 (39.3)	3 (10.7)	14 (50.0)
Household help	7 (25.0)	3 (10.7)	18 (64.3)
Rehabilitation centers	13 (46.4)	4 (14.3)	11 (39.3)
Palliative care	4 (14.3)	5 (17.9)	19 (67.9)
Sports	18 (67.1)	4 (19.8)	7 (24.1)

### Development of a quality indicator set

The process to identify and assess potential quality indicators in breast cancer care for metastatic disease took place from November 2017 to April 2018. The final indicator set was established during a phone conference of the SAB on April 26, 2018. The national evidence- and consensus-based guideline of the German Guideline Program in Oncology “Screening, Diagnosis, Therapy and Follow-up of Breast Cancer” served as standard to define high quality of health care [4, 12, 13]. From the initial pool of 14 proposed indicators, a final set of 11 indicators were selected by the members of the SAB on criteria regarding relevance of the indicators for health care/the patient and practicability in clinical routine. The final set is listed as Additional file 1: Table S1.

## Discussion

This report describes the development of a patient-centered MBC registry and its pilot testing in a defined region in Germany. Within a 9-month funding period, a registry concept for patients with MBC in the region of Main-Franconia was developed under the guidance of a steering committee, a scientific advisory board, and a patients’ representative. Cooperation with the competent Cancer Registries of Bavaria and Baden-Württemberg for record linkage and the participating hospitals for patient recruitment was established. Paper-based and electronic questionnaires were developed addressing clinical parameters as diagnosis, therapy, or progression as well as met and unmet needs of the patients and their family members. Questionnaires, patient’s acceptance of the APP and feasibility of data exchange and record linkage were successfully tested in a proof-of-concept study. A total of *n* = 31 patients with MBC gave written informed consent for participation in the proof-of-concept study.

Previous studies on the care of patients with early BC exist from the “Breast Cancer Care under Evidence-based Guidelines” (BRENDA) in Germany cohort [10, 11]. The results show that deviations from the guidelines in patients with early BC induced by the physician were associated with younger age and poorer quality of life of the patients. On the other hand, deviations based on patients’ belief are associated with older age and fears against certain treatment methods [11]. These results are not transferable to the treatment of metastasized patients, since patients with MBC receive a diagnosis of an incurable disease, which also affects patient’s family members. Information on perspectives of family members of patients with early breast cancer is missing in the BRENDA cohort. The therapy of MBC aims for a high quality of life by controlling for disease symptoms with a palliative therapy approach. Already existing registries for patients with metastasized cancer focus mainly on biomarkers, which ought to be identified as predictors for treatment response [20]. Currently, there is no registry, which depicts the structures of care and patients’ needs in an MBC collective, although a great heterogeneity in effective treatment behavior especially for metastatic cancers exists. Nevertheless, there is currently no standardized documentation in place, which describes the variations of therapy and the sequences of administered substances.

In Germany, the Cancer Registries established by law are organized on a federal basis with every state being responsible for the statutory frame. With the German National Cancer Plan (2014, §65c SGB V) a nation-wide standardized data collection was established by the German Tumour Centres Work Group (ADT) and the German Society of Population-based Cancer Registries (GEKID). This “common oncological core data set” and the specific module “mamma” (1) aim for an economic data collection, and (2) focus on clinical parameters (e.g., hormone receptors, treatment). Thus, information on appropriate care addressing patients’ needs via routinely collected patient-reported outcomes (e.g., quality of life, patients’ needs) are lacking in these registries.

The BRE-4-MED registry aims to provide a database to inform clinicians and patients as well as other stakeholders (e.g., policy makers, self-aid groups). Research outputs will be disseminated to the public and local stakeholders via press releases or other communication measures, using established regional structures, including e.g. regular presentations at public events of the CCC MF, at the local forum for women health “Franken Fortbildung Frauengesundheit” and talks at events of the local patient self-advocacy groups. To raise public awareness a website as well as a patient newsletter for reporting study results and for communicating other new developments in breast cancer care back to the patient will be developed. Health care stakeholders (e.g., scientific societies, health insurance, policy makers) will be informed about the results and quality of health care by regular reports. Participating physicians will get feedback on their guideline adherence by benchmarking reports. In addition, our data can also be accessed by external researchers for scientific analyses based on reasonable request to the steering board.

The BRE-4-MED registry concept aims to assess clinical routine care of patient with MBC. Therefore, the purpose of the concept was to make it easily to integrate by (1) keeping time to fill out the treating physician’s questionnaire (eCRF) to a minimum; (2) reducing queries by utilizing eCRF including checking conditions; and (3) carrying out a centralized follow-up at the method center. Due to these aims, we will adapt our registry concept. A central study nurse will visit the participating hospitals on a regular basis (e.g., weekly) to perform patient documentation and to fill out the eCRF.

The BRE-4-MED registry concept integrates the perspectives of patients and their family members. Our proof-of-concept study revealed that family members of patients with MBC were willing to fill out an anonymous questionnaire on burden of caregiving [18]. Since only about 10% of all BRE-4-MED participants stated to be care given by a family member, the questionnaire will be supplemented by assessment of informational and supportive needs of family members. If this adaption can increase the response rate (i.e., 50% in our pilot study) of non-caregiving family members in our registry needs to be shown.

The voluntary APP and the online questionnaire was used by about one-third of BRE-4-MED participants for documenting PROs although more than two-thirds of participants stated to own a computer or smartphone/tablet with internet access. Reasons for the low coverage might be due to the lack in skills to use web-based questionnaires since the mean age of all participants was 58 years. Furthermore, some hospitals were not able to assist participants in downloading the BRE-4-MED APP or online questionnaire due to problems with highly secured firewalls in some hospitals. Wallwiener et al. reported similar challenges on usage of electronically PROs in the “Prospective Academic translational research network for the optimization of oncological health care quality in the advanced setting” (PRAEGNANT) registry [9]. The process chosen for record linkage with both established Cancer Registries (Bavaria and Baden-Wuerttemberg) worked out without any problems. Still, for some BRE-4-MED participants (*n* = 14) no information were available in the Cancer Registries. The reason might be that the state-wide report of cancer cases is mandatory by law for Baden-Wuerttemberg since October 2011 and for Bavaria since April 2017. Thus, an assessment of all patients diagnosed with MBC in all four participating hospitals as well as all over Main-Franconia is possible on an anonymous/aggregated base. This may also reduce the effort to participating hospitals keeping a screening or recruiting sheet.

### Strengths and limitations

The main strength of our registry concept is the utilization and combination of primary and secondary data from several sources of information as well as m-health applications within one comprehensive registry. Thus, data from already established structures as the Cancer Registries and routine in-hospital and outpatient data can be combined with information reported by the patients themselves and their family members. This may lead to better information on appropriate care of patients with MBC. Furthermore, a patients’ representative was involved in the process to provide patient’s perspective on information material, recruitment procedure, and questionnaires. There are limitations of our proof-of-concept study. First, the proposed BRE-4-MED registry concept with Germany-wide standardized structures for Cancer Registries may not be applicable to international systems. Secondly, no therapy or progression occurred during the pilot phase though it is unclear how the contact and documentation of (1) a treating physician of a participating hospital or (2) a treating physician of a non-participating hospital would work out. Thirdly, the m-health solutions could not be comprehensively tested due to initial technical hurdles in some participating hospitals and due to the fact that some hospitals only offered the APP to younger participants (selection bias). Fourthly, information is collected from physicians itself in a self-report fashion, which might introduce potential bias. Fifthly, the recruitment rate was quite low (i.e., 36%), which might be caused by a short recruitment time (i.e., 3 months) due to timely restricted funding as well as the inclusion also of prevalent cases with a high disease burden. Sixthly, the retention rate for 1-month follow-up for patient’s and caregiver’s questionnaire was quite low (i.e., 77%), which might have several reasons: follow-up questionnaire was quite close to baseline assessment; no structured reminders were used to maintain a high response rate.

## Conclusion

The BRE-4-MED pilot study established the feasibility of the developed concept regarding procedures on data collection and linkage from primary and secondary data sources for an incurable patient group. Despite a low recruitment rate, which was not a core criterion on feasibility, the collection of PROMs using m-health devices is in general accepted, considering m-health is present for patients and clinicians in Germany, yet. For the next step of the study some adaptions to the procedures need to be taken into account to improve recruitment and participation rates: (1) implementing dedicated study nurse for patient recruitment also by identification of eligible patients through interdisciplinary tumor board; (2) improve visibility of registry by distribution of information and rationale of the study via self-aid groups or patient leaflets to improve recruitment; (3) establishing standardized reminder algorithms (postal and phone) to increase response rate especially over the whole follow-up period; (4) adapting the APP to allow patient keeping track of his own m-health entered data and utilize these data for doctor-patient-communication to increase acceptance of the APP for follow-up purposes.

Our registry concept provides a patient-focused assessment of met and unmet needs and clinical parameters utilizing different assessment tools (e.g., secondary data, pCRF, eCRF, App) in a longitudinal manner. Particularly, the linkage of routine data from the Cancer Registries with patient-reported outcomes allows answering relevant questions of health care service research in terms of guideline-adherent treatment, barriers of guideline implementation, and patients’ needs. Table 6 provides an overview of lessons learnt of the BRE-4-MED proof-of-concept study. We are currently seeking funding for the full implementation of our established BRE-4-MED registry concept in clinical routine care.

**Table 6:** Textbox on lessons learnt from BRE-4-MED proof-of-concept study

• Combination of primary and secondary data is feasible ○ Routinely collected data from established Cancer Registries can serve as basis and help reducing redundant data collection for physicians and can be complemented by personal PROMs follow-up information ○ Utilizing m-health in incurable cancer patients is in general accepted considering its increasing impact in the future. Adaption regarding patient’s benefit (e.g., m-health as personal diary or basis for doctor-patient-communication) will be required • Strategies for strengthening patient recruitment ○ Identification of eligible patients through interdisciplinary tumor board ○ For recruiting dedicated study nurse to improve recruitment rate ○ Standardized reminder algorithm (postal and phone) to increase response rate ○ Adaption of APP/online questionnaire to allow patient keeping track of her/his own m-health entered data and utilize these data for doctor-patient-communication ○ Improving visibility of registry by distribution of information and rationale of the study via self-aid groups or patient leaflets	

## Supplementary information

**Additional file 1: Table S1.** Proposed quality indicators for measuring guideline adherence (based on the National Guideline on Screening, Diagnosis, Therapy and Follow-up of Breast Cancer" (2017) – German Guideline Program in Oncology).

## Data Availability

The dataset used and/or analyzed during the current pilot study is available from the corresponding author on reasonable request.

## Abbreviations

ADT: German Tumour Centres Work Group
APP: Application software
BC: Breast cancer
BMBF: German Federal Ministry of Education and Research
BRE-4-MED: Breast cancer care for patients with metastatic disease
BRENDA: Breast Cancer Care under Evidence-based Guidelines
CCC MF: Comprehensive Cancer Center Mainfranken
DKG: German Cancer Society
DRKS: German Clinical Trials Register
eCRF: Electronic questionnaire
EORTC-QL: European Organisation for Research and Treatment of Cancer–Quality of Life
GEKID: German Society of Population-based Cancer Registries
ICE-B: Institute of Clinical Epidemiology and Biometry
MBC: Metastatic breast cancer
m-health: Mobile health
PAT-ID: Unique patient identifier
pCRF: Paper-based questionnaire
PRAEGNANT: Prospective Academic translational research network for the optimization of oncological health care quality in the advanced setting
PROMs: Patient-reported outcome measures
PROs: Patient-reported Outcomes
SAB: Scientific advisory board
ZTM: Zentrum für Telemedizin Bad Kissingen (Center for telemedicine)

## References

[1] Jamtvedt G, Young JM, Kristoffersen DT, et al. Audit and feedback: effects on professional practice and health care outcomes. The Cochrane database of systematic reviews 2006(2):Cd000259. 10.1002/14651858.CD000259. pub2[published Online First: Epub Date]|. 10.1002/14651858.CD000259.pub2 16625533

[2] Ferlay J, Soerjomataram I, Dikshit R (2015). Cancer incidence and mortality worldwide: sources, methods and major patterns in GLOBOCAN 2012. Int J Cancer.

[3] Chung CT, Carlson RW (2003). Goals and objectives in the management of metastatic breast cancer. The oncologist.

[4] Leitlinienprogramm Onkologie (Deutsche Krebsgesellschaft, Deutsche Krebshilfe, AWMF). S3-Leitlinie Früherkennung, Diagnose, Therapie und Nachsorge des Mammakarzinoms. Version 4.1. 2017. AWMF Registernummer: 032-045OL, 2017.

[5] (NICE) NIfHaCE. Early and locally advanced breast cancer: diagnosis and management. Secondary EARLY and locally advanced breast cancer: diagnosis and management 2018. https://www.nice.org.uk/guidance/ng101.

[6] Burstein Harold J., Lacchetti Christina, Anderson Holly, Buchholz Thomas A., Davidson Nancy E., Gelmon Karen A., Giordano Sharon H., Hudis Clifford A., Solky Alexander J., Stearns Vered, Winer Eric P., Griggs Jennifer J. (2019). Adjuvant Endocrine Therapy for Women With Hormone Receptor–Positive Breast Cancer: ASCO Clinical Practice Guideline Focused Update. Journal of Clinical Oncology.

[7] Mestres JA, iMolins AB, Martinez LC (2017). Defining the optimal sequence for the systemic treatment of metastatic breast cancer. Clin Trans Oncol.

[8] Rocque Gabrielle B., Williams Courtney P., Kenzik Kelly M., Jackson Bradford E., Azuero Andres, Halilova Karina I., Ingram Stacey A., Pisu Maria, Forero Andres, Bhatia Smita (2018). Concordance with NCCN treatment guidelines: Relations with health care utilization, cost, and mortality in breast cancer patients with secondary metastasis. Cancer.

[9] Wallwiener M, Heindl F, Brucker SY (2017). Implementation and feasibility of electronic patient-reported outcome (ePRO) Data Entry in the PRAEGNANT Real-Time Advanced and Metastatic Breast Cancer Registry. Geburtshilfe und Frauenheilkunde.

[10] Schwentner L, Van Ewijk R, Kuhn T (2016). Exploring patient- and physician-related factors preventing breast cancer patients from guideline-adherent adjuvant chemotherapy-results from the prospective multi-center study BRENDA II. Support Care Cancer.

[11] Stuber T, van Ewijk R, Diessner J (2017). Which patient- and physician-related factors are associated with guideline adherent initiation of adjuvant endocrine therapy? Results of the prospective multi-centre cohort study BRENDA II. Breast Cancer (Tokyo, Japan).

[12] Wöckel A, Festl J, Stüber T (2018). Interdisciplinary screening, diagnosis, therapy and follow-up of breast cancer. Guideline of the DGGG and the DKG (S3-Level, AWMF Registry Number 032/045OL, December 2017) – Part 2 with recommendations for the therapy of primary, recurrent and advanced breast cancer. Geburtshilfe und Frauenheilkunde.

[13] Wockel A, Festl J, Stuber T (2018). Interdisciplinary screening, diagnosis, therapy and follow-up of breast cancer. Guideline of the DGGG and the DKG (S3-Level, AWMF Registry Number 032/045OL, December 2017) - Part 1 with recommendations for the screening, diagnosis and therapy of breast cancer. Geburtshilfe und Frauenheilkunde.

[14] Geraedts M, Selbmann HK, Ollenschlaeger G (2003). Critical appraisal of clinical performance measures in Germany. Int J Qual Health Care.

[15] Measuring and improving quality of care: a report from the american heart Association/American college of cardiology first scientific forum on assessment of healthcare quality in cardiovascular disease and stroke. Stroke. 2000;31(4):1002–12. 10.1161/01.str.31.4.1002 10754017

[16] Haas K, Stangl S, Steigerwald F (2019). Development of evidence-based quality indicators for deep brain stimulation in patients with Parkinson’s disease and first year experience of implementation of a nation-wide registry. Parkinsonism Relat Disord.

[17] Lowe B, Wahl I, Rose M (2010). A 4-item measure of depression and anxiety: validation and standardization of the Patient Health Questionnaire-4 (PHQ-4) in the general population. J Affect Disord.

[18] Given CW, Given B, Stommel M (1992). The caregiver reaction assessment (CRA) for caregivers to persons with chronic physical and mental impairments. Research in nursing & health.

[19] Tieman JJ, Swetenham K, Morgan DD, et al. Using telehealth to support end of life care in the community: a feasibility study. BMC Palliative Care. 2016;15(1):–94. 10.1186/s12904-016-0167-7 [published Online First: Epub Date]|. 10.1186/s12904-016-0167-7 PMC5114812 27855681

[20] Hartkopf AD, Huober J, Volz B (2018). Treatment landscape of advanced breast cancer patients with hormone receptor positive HER2 negative tumors - data from the German PRAEGNANT breast cancer registry. Breast (Edinburgh, Scotland).

