# Supplementary material for: Development and proof-of-concept of a multicenter, patient-centered cancer registry for breast cancer patients with metastatic disease—the “Breast cancer care for patients with metastatic disease” (BRE-4-MED) registry
Source: Pilot Feasibility Stud. 2020 Feb 4;6:11. doi: 10.1186/s40814-019-0541-3 (PMC7001276; doi:10.1186/s40814-019-0541-3)
Supplement: Supplementary file 1 — Additional file 1: Table S1. Proposed quality indicators for measuring guideline adherence (based on the National Guideline on Screening, Diagnosis, Therapy and Follow-up of Breast Cancer" (2017) – German Guideline Program in Oncology). [file 40814_2019_541_MOESM1_ESM.docx]

Additional Data - Table 1: Proposed quality indicators for measuring guideline adherence (based on the National Guideline on Screening, Diagnosis, Therapy and Follow-up of Breast Cancer" (2017) – German Guideline Program in Oncology)

| **BRE-4-MED quality indicators (process)** | **Background** | **Numerator/Denominator** |
| --- | --- | --- |
| Histological confirmation of metastases | A biopsy of metastases should be undertaken to assess expression profile | **Numerator:** Patients with metastatic breast cancer confirmation of metastasis  **Denominator:** Patients with metastatic breast cancer |
| Endocrine first-line therapy | Endocrine (-based) therapy should be therapy of choice in patients with positive hormone receptor status and negative HER2 status. Endocrine therapy should not be administered where a fast remission needs to be reached | **Numerator:** Patients with hormone receptor positive metastatic breast cancer, who undergo endocrine therapy as first-line treatment  **Denominator:** Patients with hormone receptor positive metastatic breast cancer |
| Chemo and endocrine combined therapy | A combined therapy should not be recommended as the increased remission rate of this therapy combination does not weigh out the increased toxicity without a prolonging in progression-free or overall survival | **Numerator:** Patients with hormone receptor positive metastatic breast cancer, who undergo chemo and endocrine combined therapy  **Denominator:** Patients with hormone receptor positive metastatic breast cancer |
| Chemo and monoclonal antibody combined therapy in first-line therapy | As first-line treatment in patients with HER2-positive metastatic breast cancer a combination of a taxan and Docetaxel / Trastuzumab / Pertuzumab is recommended | **Numerator:** Patients with HER2-positive metastatic breast cancer, who are treated with (Docetaxel/Trastuzumab/Pertuzumab) as first-line treatment  **Denominator:** Patients with HER2-positive metastatic breast cancer |
| Treatment with trastuzumab emtansin (T-DM1) in second-line | Patients with HER2-postive metastatic breast cancer should receive T-DM1 as second-line therapy | **Numerator:** Patients with HER2-positive metastatic breast cancer, who are treated with TDM-1 therapy as second-line treatment  **Denominator:** Patients with HER2-positive metastatic breast cancer |
| Recommendation for surgery/radiation by an interdisciplinary tumour board | Indication for an operative or ablative therapy should be discussed in an interdisciplinary tumour board | **Numerator:** Patients with metastatic breast cancer, whose treatment strategy with surgery or radiation was discussed in an interdisciplinary tumour board  **Denominator:** Patients with metastatic breast cancer, who undergo surgery or radiation |
| Pleurodesis | Patients with malignant pleural effusion should be offered a pleurodesis | **Numerator:** Patients with metastatic breast cancer and malignant pleural effusion, who undergo pleurodesis  **Denominator:** Patients with metastatic breast cancer and malignant pleural effusion |
| Information about psycho-oncological therapy | Patients and their relatives should be informed during the course of breast cancer about psycho-oncological support | **Numerator:** Patients with metastatic breast cancer, who got offered psycho-oncological therapy  **Denominator:** Patients with metastatic breast cancer |
| Psycho-oncological therapy | Patients should undergo psycho-oncological treatment | **Numerator:** Patients with metastatic breast cancer, who undergo psycho-oncological therapy  **Denominator:** Patients with metastatic breast cancer |
| Recommendation for palliative care by a specialist for palliative care | All patients with metastatic breast cancer should be offered palliative care (according to the individual therapy and life goals of the patient) | **Numerator:** Patients with metastatic breast cancer, who undergo intravenous chemotherapy and who got recommendation for consultation by a specialist in palliative care  **Denominator:** Patients with metastatic breast cancer, who undergo intravenous chemotherapy |
| Supportive physiotherapy | All patients with metastatic breast cancer should be offered supportive physiotherapy | **Numerator:** Patients with metastatic breast cancer, who undergo physiotherapy as part of breast cancer treatment  **Denominator:** Patients with metastatic breast cancer |
